# Supplementary material for: Systematic Review of the Genus Nalepa Reitter, 1887 (Coleoptera, Tenebrionidae, Blaptinae, Blaptini) from the Tibetan Plateau, with Description of Six New Species and Two Larvae
Source: Insects. 2022 Jun 29;13(7):598. doi: 10.3390/insects13070598 (PMC9316563; doi:10.3390/insects13070598)
Supplement: Supplementary file 1 [file insects-13-00598-s001.zip › insects-1783957-supplementary.pdf]

Table S1. List of specimens used in this study with the corresponding accession number.

No	Species	Sampling locality	Elevation (m)	Date of collection	Collector(s)	Preservation	Accession number / - lacing<br>COI (1490, 2198) / COI (2183, 3014) / COII / Cytb / 16S / 28S
QHN01	*Nalepa cylindracea*	Gesang, Zadoi, Qinghai, China	4043	2019-VII-25	X. Bai *et al.*	Ethanol	ON827513/ON827499 /ON856151/---------/ON818489
TiBN01	*Nalepa cylindracea*	Gyidoi, Riwoqê, Xizang, China	3837	2019-VII-27	X. Bai *et al.*	Ethanol	ON827515/ON827501/---------/---------/ON818464/ON818491
QHN02	*Nalepa cylindracea*	Nangqên, Qinghai, China	n/a	2014-VII-29	Zh. Zhou	Dried	n/a
QHN03	*Nalepa cylindracea*	Oyala pass, Nangqên, Qinghai, China	4432	2019-VII-27	X. Bai *et al.*	Ethanol	ON827516/ON827502/ON856154/ON856230/ON818465/ON818492
TiBN02-larva	*Nalepa cylindracea*	Ya’ ngan, Baqen, Xizang, China	4135	2018-VIII-21	X. Bai *et al.*	Ethanol	ON827517/ON827503/ON856155/ON856232/ON818466/ON818493
TiBN03	*Nalepa cylindracea*	Ya’ ngan , Baqen, Xizang, China	4135	2018-VIII-21	X. Bai *et al.*	Ethanol	ON827525/ON827507/ON856159/ON856250/ON818484/ON818513
TiBN04	*Nalepa cylindracea*	G214 road, Mangkang, Xizang, China	2689	2021-VII-13	X. Li *et al.*	Ethanol	ON827518/ON827504/ON856156/ON856233/ON818467/ON818494
TiBN05	*Nalepa cylindracea*	Qu’ nyido, Jomda, Xizang, China	4050	2016-VIII-8	X. Li *et al.*	Ethanol	---------/ON827490/ON856168/ON856239/ON818468/ON818501
TiBN06	*Nalepa cylindracea*	Qu’ nyido, Jomda, Xizang, China	3924	2016-VIII-9	X. Li *et al.*	Ethanol	---------/ON827491/---------/ON856240/ON818473/ON818502
TiBN07	*Nalepa cylindracea*	Qu’ nyido, Jomda, Xizang, China	3857	2009-VII-20	G. Ren *et al.*	Dried	n/a
TiBN08	*Nalepa cylindracea*	Toba, Chamdo, Xizang, China	4017	2016-VIII-9	X. Li *et al.*	Ethanol	ON827521/ON827497/ON856169/ON856248/ON818481/ON818510
TiBN09	*Nalepa cylindracea*	Gyanbê, Gonjo, Xizang, China	3758	2016-VIII-8	X. Li *et al.*	Ethanol	ON827522/ON827492/ON856170/ON856241/ON818474/---------
TiBN10	*Nalepa cylindracea*	Lhatog, Chamdo, Xizang, China	3724	2016-VIII-9	X. Li *et al.*	Ethanol	ON827523/ON827493/ON856171/ON856242/ON818475/ON818503
TiBN11	*Nalepa cylindracea*	Zom La shan, Mangkang, Xizang, China	4186	2016-VIII-12	X. Li *et al.*	Ethanol	---------/ON827508/ON856160/ON856251/ON818485/ON818514
QHN05	*Nalepa cylindracea*	Aangsai, Zadoi, Qinghai, China	n/a	2014-VII-3	Zh. Zhou	Dried	n/a
QHN04-Larva	*Nalepa cylindracea*	Saiqu, Zadoi, Qinghai, China	4045	2012-VII-22	G. Ren *et al.*	Dried	---------/ON827496/---------/ON856245/ON818478/ON818506
QHN06	*Nalepa*	Longbao, Yushu,	4209	2012-VII-23	G. Ren *et*	Dried	n/a

No	Species	Sampling locality	Elevation (m)	Date of collection	Collector(s)	Preservation	Accession number / - lacing COI (1490, 2198) / COI (2183, 3014) / COII / Cytb / 16S / 28S
	cylindracea	Qinghai, China			*al.*		
QHN07	*Nalepa cylindracea*	Longbao, Qinghai, China	4361	2012-VII-23	G. Ren *et al.*	Dried	n/a
QHN08	*Nalepa cylindracea*	Chenwen, Chindu, Qinghai, China	3996	2009-VII-17	G. Ren *et al.*	Dried	n/a
QHN09	*Nalepa cylindracea*	Sahuteng, Zadoi, Qinghai, China	4292	2012-VII-22	G. Ren *et al.*	Dried	n/a
QHN10	*Nalepa cylindracea*	Lixin, Zhidoi, Qinghai, China	4144	2012-VII-23	G. Ren *et al.*	Dried	n/a
QHN11	*Nalepa cylindracea*	Dake, Zhidoi, Qinghai, China	4117	2012-VII-23	G. Ren *et al.*	Dried	n/a
TiBN12	*Nalepa cylindracea*	Xoji la Shan, Jomda, Xizang, China	4240	2014-VII-27	G. Ren *et al.*	Dried	n/a
SCN01	*Nalepa quadrata* sp. n.	Le·an, Xinlong, Sichuan, China	4015	2016-VIII-14	X. Li *et al.*	Ethanol	---------/ON827494/ON856173/ON856243/ON818476/ON818504
SCN02	*Nalepa quadrata* sp. n.	G350 road, Luhuo, Sichuan, China	3094	2021-VII-17	X. Li *et al.*	Ethanol	ON827519/ON827505/ON856157/---------/ON818468/ON818495
SCN03	*Nalepa quadrata* sp. n.	Simu, Luhuo, Sichuan, China	3180	2016-VIII-3	X. Li *et al.*	Ethanol	---------/ON827509/ON856161/ON856252/ON818486/ON818515
SCN04-Larva	*Nalepa quadrata* sp. n.	Simu, Luhuo, Sichuan, China	3180	2016-VIII-3	X. Li *et al.*	Ethanol	---------/ON827510/ON856162/ON856253/ON818487/ON818516
SCN05	*Nalepa xinlongensis* sp. n	Mari, Xinlong, Sichuan, China	4028	2016-VIII-14	X. Li *et al.*	Ethanol	ON827524/ON827498/ON856172/ON856249/ON818482/ON818511
TiBN13	*Nalepa xinlongensis* sp. n	Jitang, Chagyab, Xizang, China	3554	2016-VIII-10	X. Li *et al.*	Ethanol	n/a
SCN06	*Nalepa polita* sp. n	Rinda, Luhuo, Sichuan, China	3092	2016-VIII-3	X. Li *et al.*	Ethanol	---------/ON827487/ON856164/ON856234/ON818469/ON818497
SCN07	*Nalepa polita* sp. n	Yagra, Garzê, Sichuan, China	3670	2016-VIII-5	X. Li *et al.*	Ethanol	---------/ON827511/---------/---------/ON856236/ ON818498
SCN08	*Nalepa polita* sp. n	Yiniu, Sêrxü, Sichuan, China	3937	2009-VII-18	G. Ren *et al.*	Dried	n/a
SCN09	*Nalepa polita* sp. n	Sêrxü Si, Sêrxü, Sichuan, China	4113	2009-VII-17	G. Ren *et al.*	Dried	n/a
TiBN14	*Nalepa polita* sp. n	Latog, Qamdo, Xizang, China	n/a	2011-VII-27	G. Ren *et al.*	Dried	n/a

No	Species	Sampling locality	Elevation (m)	Date of collection	Collector(s)	Preservation	Accession number / - lacing
							COI (1490, 2198) / COI (2183, 3014) / COII / Cytb / 16S / 28S
SCN10	*Nalepa polita* sp. n	Chowa, Dêgê, Sichuan, China	n/a	2011-VII-26	G. Ren *et al.*	Dried	---------/---------/ON856163/ON856254/ON818488/ON818517
SCN11	*Nalepa acuminata* sp. n	Jinsha, Baiyü, Sichuan, China	2980	2016-VIII-6	X. Li *et al.*	Ethanol	---------/---------/---------/ON856246/ON818479 /ON818508
SCN12	*Nalepa acuminata* sp. n	Hepo, Baiyü, Sichuan, China	3000	2016-VIII-6	X. Li *et al.*	Ethanol	---------/---------/---------/---------/ON856235/ON818496
TiBN15	*Nalepa yushuensis* sp. n	Chaiwei, Qamdo, Xizang, China	3403	2018-VIII-23	X. Bai *et al.*	Ethanol	---------/ON827506/ON856158/ON856255/ON818483/ON818512
QHN12	*Nalepa yushuensis* sp. n	Jyêgu, Yushu, Qinghai, China	4008	2019-VII-24	X. Bai *et al.*	Ethanol	ON827514/ON827500/ON856152/ON856231/ON818463/ON818490
QHN13	*Nalepa yushuensis* sp. n	Shanglaxiu, Yushu, Qinghai, China	4227	2012-VII-21	G. Ren *et al.*	Dried	---------/ON827495/---------/ON856244/ON818477/ON818505
SCN14	*Nalepa xinlongensis* sp. n	Baiya, Dêgê, Sichuan, China	3021	2016-VIII-6	X. Li *et al.*	Ethanol	n/a
TiBN16	*Nalepa ovalifolia* sp. n	Gyamda, Jomda, Xizang, China	3630	2016-VIII-8	X. Li *et al.*	Ethanol	---------/ON827488/ON856165/ON856237/ON818470/ON818499
SCN15	*Nalepa ovalifolia* sp. n	Xindu, Luhuo, Sichuan, China	3194	2016-VIII-6	X. Li *et al.*	Ethanol	ON827520/ON827489/ON856167/ON856238/ON818471/ON818500
SCN16	*Nalepa ovalifolia* sp. n	Gongya, Dêgê, Sichuan, China	3120	2016-VIII-6	X. Li *et al.*	Ethanol	---------/---------/ON856166/ON856247/ON818480/ON818509
TiBN17	*Nalepa ovalifolia* sp. n	Kargang, Jomda, Xizang, China	3700	2016-VIII-08	X. Li *et al.*	Ethanol	n/a
TiBN18	*Nalepa ovalifolia* sp. n	Tangpu, Jomda, Xizang, China	3293	2016-VIII-07	X. Li *et al.*	Ethanol	n/a
SCN17	*Nalepa ovalifolia* sp. n	Ronggai, Baiyü, Sichuan, China	3120	2016-VIII-5	X. Li *et al.*	Ethanol	n/a
TiBN19	*Nalepa ovalifolia* sp. n	Yi· lhung, Jomda, Xizang, China	4164	2016-VIII-9	X. Li *et al.*	Ethanol	n/a
